# Evaluation of Noninvasive Adjuncts for Early Detection of Oral Cancer in Oral Potentially Malignant Disorders and Development of Risk-Based Management Strategies: Protocol for a Prospective Longitudinal Study

**DOI:** 10.2196/66285

**Published:** 2025-05-28

**Authors:** Ruchika Gupta, Apurva Ratnu, Shalini R Gupta, Hariprakash Hadial, Lucky Singh, Prashant Kumar Singh, Shalini Singh

**Affiliations:** 1 ICMR-National Institute of Cancer Prevention and Research Noida India; 2 Associate Professor, Faculty of Biological Sciences Academy of Scientific and Innovative Research Ghaziabad India; 3 Niramay Charitable Trust Gandhinagar India; 4 Centre for Dental Education and Research All India Institute of Medical Sciences New Delhi India; 5 Indian Council of Medical Research New Delhi India; 6 Associate Professor, Faculty of Mathematical and Information Sciences Academy of Scientific and Innovative Research Ghaziabad India; 7 Associate Professor, Faculty of Medical Research Academy of Scientific and Innovative Research Ghaziabad India

**Keywords:** oral potentially malignant disorder, noninvasive adjunct, toluidine blue, autofluorescence, risk stratification, management

## Abstract

**Background:**

Oral potentially malignant disorders (OPMDs) constitute the most important precursors of oral cancer. Histopathological examination of a biopsy from a clinically suspicious lesion is still the gold standard for the diagnosis of oral cancer. Adjunctive techniques such as autofluorescence, toluidine blue (TB), and others have been evaluated among high-risk individuals such as chronic tobacco chewers or in patients with suspicious lesions. However, evaluation of these noninvasive adjunctive techniques has not been performed in primary health care settings. Since the first point-of-contact of individuals living in rural and semiurban areas are the primary health care workers, evaluation of these noninvasive adjuncts is likely to assist and strengthen the population-wide oral cancer screening in high-burden countries such as India.

**Objective:**

This prospective longitudinal study aims to evaluate the noninvasive adjuncts in oral cancer screening in the field settings, specifically in detecting foci of oral cancers in various OPMDs.

**Methods:**

After staff recruitment and training, we shall conduct oral cancer screening camps in the community for the recruitment of individuals with OPMDs after obtaining informed consent. All patients with OPMD shall undergo further screening via autofluorescence and TB staining for detection of lesions suspicious of oral cancer. Sensitivity, specificity, and negative and positive predictive values of these adjunctive techniques (autofluorescence and TB) in the detection of oral cancer shall be calculated using biopsy as the gold standard. In addition, this study will also focus on the validation of the 2022 consensus guidelines on risk-based stratification and appropriate management protocols for the OPMDs at the primary and referral health care centers. Our primary outcome is the diagnostic use of autofluorescence and TB in oral cancer detection among OPMDs as well as the robustness of the risk-based management protocols for these patients.

**Results:**

Participant recruitment has been initiated at all sites. Staff recruitment and training in the oral visual examination have been conducted. Procurement of the autofluorescence device is in progress. All the study sites have begun conducting oral screening camps.

**Conclusions:**

The results of this study shall provide robust evidence for the diagnostic use of autofluorescence and TB staining in early oral cancer detection among patients with OPMD. The use of these noninvasive adjuncts by primary health care providers can significantly improve oral cancer screening in our country. The validation of risk-based stratification and management of patients with OPMD shall assist in the refinement of the national guidelines for these interventions. This study has been approved by the respective ethics committee of ICMR-National Institute of Cancer Prevention and Research and the collaborating institutes. The findings of this study shall be disseminated through scientific publications in peer-reviewed journals as well as meetings with the concerned stakeholders at the district and state health departments.

**International Registered Report Identifier (IRRID):**

DERR1-10.2196/66285

## Introduction

Oral cancer constitutes the second most common cancer in India contributing 10.3% of incident cancer cases [1]. A large majority (80%) of oral cancers develop from oral potentially malignant disorders (OPMDs) [2]. Risk factors such as tobacco chewing, alcohol use, areca nut chewing, and infection by human papillomavirus are common among OPMDs and oral cancer [3]. Other common factors include chronic oral trauma from ill-fitting dentures or broken teeth, chronic inflammation, microbiome alterations, systemic sclerosis, genetic dysregulation of DNA metabolism, and nutritional deficiencies [4].

OPMDs are a heterogeneous group of diseases with distinctive histopathological entities including oral leukoplakia (OL), erythroplakia, erythroleukoplakia, oral lichen planus (OLP), and oral submucous fibrosis (OSMF) [4]. The risk of malignant transformation differs among the different entities, ranging from low in homogenous leukoplakia to high for erythroleukoplakia, proliferative verrucous leukoplakia, OSMF, and very high in EP [5].

Oral cancer control and prevention efforts have been hindered by the lack of awareness and the attendant late diagnosis in the majority of cases, especially among high-risk populations including tobacco, alcohol, or areca nut users [6]. The gold standard for OPMD and oral cancer diagnosis is oral visual examination (OVE) with histopathological examination of the suspicious oral lesions [4]. However, in high-burden and resource-constrained countries such as India, the availability of health care providers trained in OVE and performing tissue biopsies as well as pathologists who would examine these biopsies at the primary health care facilities are far from ideal. As per the NHFS-5 (National Family Health Survey 5; 2019-21), only 0.9% of females and 1.2% of males have ever undergone an oral examination for oral cancer. OVE is not able to detect foci of malignant transformation within the OPMD lesions [7]. This low sensitivity of OVE in detecting dysplastic lesions exerts an undue burden on health care facilities for oral biopsy [4]. Hence, adjunctive tests are required to help detect lesions that are likely to have a malignant focus within them, thereby indicating when and where to take a biopsy. Two such noninvasive adjunctive tests are autofluorescence and toluidine blue (TB) staining. Autofluorescence makes use of the change in tissue fluorescence in dysplastic foci. Normally, tissue autofluorescence is produced due to natural fluorophores such as collagen, tryptophan, elastin, keratin, etc, which can be excited by short wavelengths and emit light of longer wavelengths [8]. In OPMDs and oral cancers, a change in the fluorophore concentration leads to alterations in light scattering and absorption by tissues [8]. TB or tolonium chloride is a metachromatic dye staining nucleic acids (DNA and RNA). This property of TB is used in the rapid on-site evaluation of fine needle aspiration cytology, especially in visceral organ nodules [9]. Studies have shown the sensitivity and specificity of TB as 92.6 and 67.9%, respectively with an accuracy of 80% for the detection of oral cancer [10]. A recently published systematic review and bivariate meta-analysis showed the diagnostic odds ratio as 7.017 with sensitivity and specificity of 73% and 69% [11]. However, the majority of the studies on these noninvasive adjuncts have been conducted in selected populations or patients attending hospitals for various oral lesions.

The therapeutic strategies currently being practiced for OPMD are empirical. A set of consensus guidelines have been published in 2022 for the management of OPMDs [12]. These guidelines have not been evaluated yet in the field settings. Since the first point of contact for the majority of the Indian population is the primary health center, the optimal evaluation and management strategies for various OPMDs need to be delineated for future implementation.

Hence, the main aim of the study is to evaluate the effectiveness of noninvasive adjuncts in the early detection of oral cancer among cases of oral potentially malignant disorders by primary health care providers. In addition, a secondary objective is to validate the risk-based management protocols for patients with OPMDs. The research questions of this study are as follows:

1. Can autofluorescence or TB improve early diagnosis of oral cancer among OPMDs as compared with OVE alone?
2. Can standardized treatment protocols based on risk-based stratification of patients with OPMD improve treatment outcomes?

## Methods

### Overview

This is an observational prospective longitudinal study.

#### Recruitment

The study is ongoing at 2 sites, Gautam Budh Nagar district in Uttar Pradesh in collaboration with the Centre for Dental Education and Research, New Delhi, and Gandhinagar in Gujarat, India. For participant recruitment, community-based outreach camps for oral cancer screening shall be organized in collaboration with the concerned district authorities and local community health workers. The staff shall be trained in OVE and the use of noninvasive screening adjuncts. Individuals with oral lesions suggestive of one of the OPMDs shall be recruited in the study after obtaining informed consent.

#### Inclusion Criteria

Individuals diagnosed with OPMDs on OVE as per the World Health Organization clinical diagnostic criteria [13] and categorized clinically as OL, EP, OLP, OSMF, tobacco pouch keratosis, smoker’s palate, and proliferative verrucous leukoplakia. For the assessment of diagnostic accuracy of the noninvasive adjuncts, 2 types of controls shall be included, a group of healthy adults with a history of tobacco or areca nut use and normal oral mucosa on OVE and a group of healthy adults with no tobacco or areca nut or alcohol habits and normal oral mucosa.

#### Exclusion Criteria

Individuals shall be excluded from this study if they have complete trismus preventing adequate mouth opening to allow OVE or the noninvasive adjuncts, history of cancer in the oral cavity, and are unwilling to participate in the study.

#### Study Procedures

**Figure 1:**
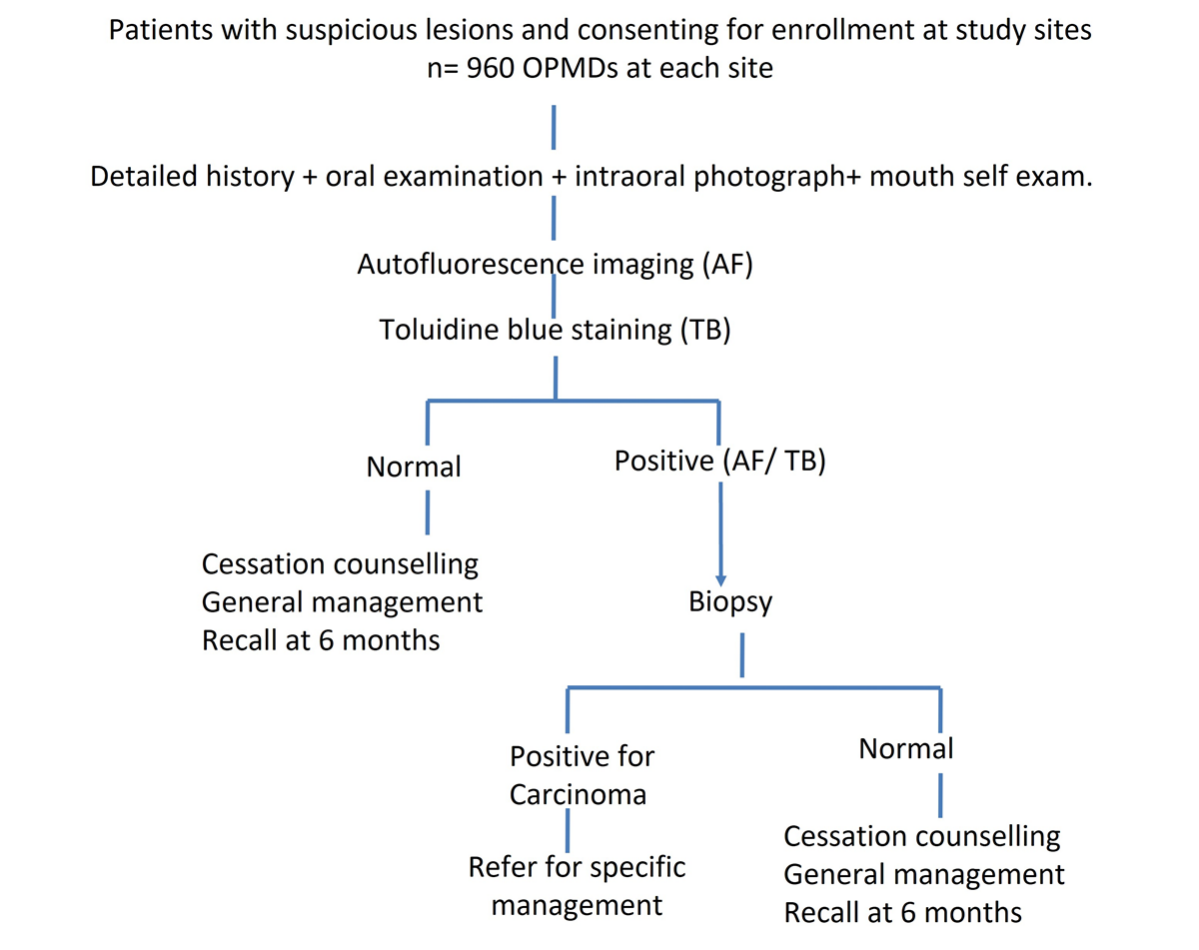
Flowchart depicting the study methodology. AF: autofluorescence; ICMR: Indian Council for Medical Research; OPMD: oral potentially malignant disorder; TB: toluidine blue.

Following inclusion into the study, the sociodemographic profile of the individuals along with health behavior and clinic-pathological characteristics shall be recorded in a predesigned proforma (Multimedia Appendix 1). OVE shall be performed in all patients and intraoral photographs of the oral mucosa shall be captured in a standardized format. Screening adjuncts shall be used on all OPMDs in the following order: autofluorescence followed by TB staining, as depicted in Figure 1. Images of each method shall be captured before advancing to the next technique. The procedures of these 2 adjuncts are described in the subsequent sections. All participants shall also be trained in mouth self-examination techniques. Individuals with tobacco, areca nut, or alcohol habits shall be offered behavioral counseling for cessation of the respective substance use. Patients with OPMD shall be asked to follow up after 15 days for routine investigations before the oral biopsy. Using oral biopsy as the gold standard, the sensitivity and specificity of autofluorescence and TB for oral cancer detection shall be computed.

Patients with histopathological evidence of moderate to vigorous dysplasia or malignancy shall be referred to the collaborating hospitals for further management as per the risk-stratified protocols discussed later. Patients not reporting for follow-up shall be contacted via home visits or telephonically. In total, 5 such attempts shall be made before considering the participant as lost to follow-up.

#### Procedure for the Use of Autofluorescence

Following the conventional OVE and intraoral photograph of the abnormal lesion, autofluorescence light from the selected device shall be used for an oral examination, focusing on the abnormal area seen on OVE. The operator and the patient shall wear safety glasses during the procedure. An intraoral photograph shall be taken with the autofluorescence light.

For visual autofluorescence, a VELscope (Visually Enhanced Lesion scope) or similar equipment that uses a fluorescent light with a wavelength of 400-460 nm shall be used. Using this wavelength of light, normal mucosa appears pale green while abnormal tissue looks dark, since the quantity and quality of fluorophores are reduced [14].

#### Use of Vital Staining With TB

In this technique, any abnormal area shall be first swabbed with 1% acetic acid for 20 seconds to remove the food debris and mucus followed by the application of 1% TB for 20 seconds on the abnormal area seen by OVE or autofluorescence. This shall be followed by swabbing with 1% acetic acid for 20 seconds to remove the extra retained TB and an intraoral photograph of the stained area shall be captured.

### Risk-Based Management of OPMDs

OPMDs shall be classified into low-risk and high-risk as follows (Textbox 1).

**Risk-based classification of oral potentially malignant disorders.**

- Low-risk oral potentially malignant disorder (OPMD)
  Mild to moderate oral submucous fibrosis.
  Nonerosive oral lichen planus.
  Homogenous oral leukoplakia.
  Tobacco pouch keratosis.
  Smoker’s palate.
  Mild dysplasia.
- High-risk OPMD
  Severe oral submucous fibrosis.
  Erosive oral lichen planus.
  Nonhomogeneous oral leukoplakia.
  Proliferative verrucous leukoplakia.
  Erythroplakia.
  Oral lichenoid lesions.
  Moderate to vigorous dysplasia.
- All patients with OPMDs and those with any one or more of tobacco, areca nut, or alcohol habits with normal-appearing mucosa shall be offered cessation counseling.
- Other general measures for all patients with OPMD shall include removal of chronic irritation and infections, diet advice with antioxidant-rich foods, adequate control of systemic diseases, and training on self-examination of the mouth.
- Specific medical or surgical management based on the type and severity of OPMD shall be undertaken.

### Statistical Analysis

Demographic and clinical data including risk factors for OPMD, clinical examination features, findings of OVE, autofluorescence, TB, and histopathological features shall be recorded in a predesigned proforma.

The association between risk factors and the presence of OPMD or oral cancer will be studied. Descriptive statistics will be applied to qualitative and quantitative data, as applicable. Performance characteristics (sensitivity, specificity, positive, and negative predictive value) shall be calculated for autofluorescence and TB using histopathology of oral biopsy as the gold standard. This shall be compared with an OVE.

### Sample Size

Considering the sensitivity and specificity of autofluorescence and TB as 0.89 and 0.8 and 0.88 and 0.67, respectively, and CI as 0.07 (width of the confidence interval), the required number of individuals with and without the disease is:



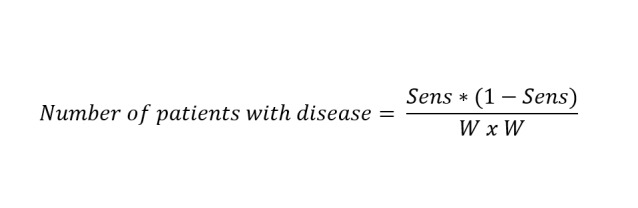





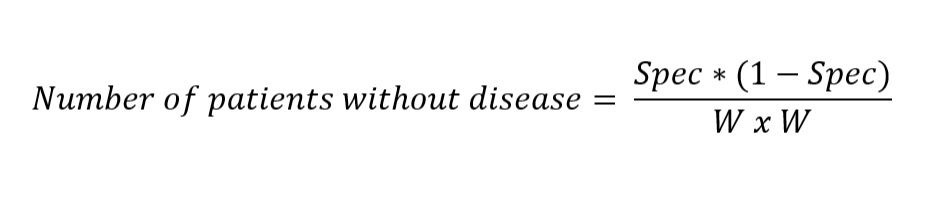



This gives the required sample size of 80 individuals with disease and 131 without disease.

Considering the positivity rate of oral biopsy in clinically suspected individuals as 25%, a total of 320 participants shall be required to undergo oral biopsy to achieve the required number. We intend to increase this number by 50% accounting for the denial or opt-out from oral biopsy, taking this number to 480 patients with OPMD requiring biopsy.

### Ethical Considerations

The study involves human participants and adheres to all applicable ethical standards and procedures. The study has been approved by the Institutional Ethics Committee, ICMR-NICPR, Noida, and the respective ethics committees of the collaborating institutes (NICPR/Ethics/2023, IEC-167/11.04.2023; SIMS/SIMS-EC/RP02/80/2024). An informed consent form shall be used in the study. For data management, the study shall implement a secure system to protect patient confidentiality. Only authorized individuals involved in the study with legitimate access to the data shall be permitted to do so. All procedures in the study shall follow the Declaration of Helsinki.

## Results

**Figure 2:**
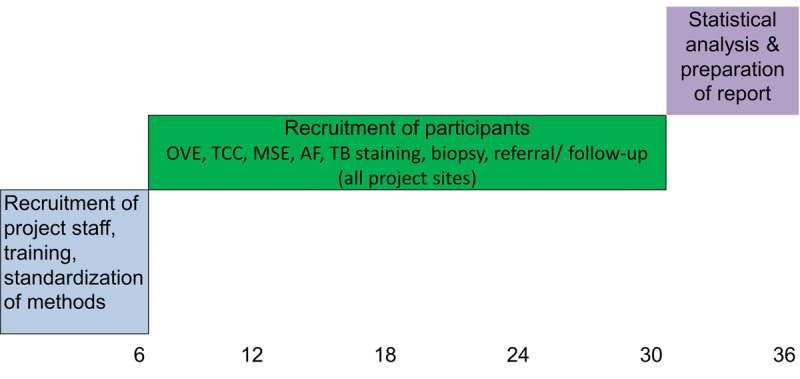
Timelines with achievable targets. AF: autofluorescence; MSE: mouth self-examination; OVE: oral visual examination; TB: toluidine blue; TCC: tobacco cessation counseling.

The study is being supported by a grant from the Indian Council of Medical Research under its Intramural Research Projects (No. 79/3/IIRP-NICPR/Onco/2023-NCD-III). Funds for the study were received in February 2024. The timeline of the study is shown in Figure 2. Participant recruitment has been initiated at all sites. Staff recruitment and training in OVE has been conducted. Procurement of the autofluorescence device is in progress. All the study sites have begun conducting oral screening camps. As of date, a total of 38 patients with OPMD have been recruited in the study. However, patient recruitment shall be enhanced once the autofluorescence device is available at the study sites. The study results shall be published in a peer-reviewed indexed scientific journal and presented at national and international conferences.

## Discussion

### Principal Findings

This study will provide robust scientific evidence on the sensitivity, specificity, and positive predictive value of diagnostic adjuncts for early diagnosis of oral cancer among patients with OPMD. A recently published review of systematic reviews reported a variable sensitivity and specificity of autofluorescence techniques for the detection of OPMD and oral cancer. Hence, the authors recommended that autofluorescence or TB staining could not replace conventional oral examination or oral biopsy [15]. However, the usefulness of these noninvasive techniques as “adjuncts” to be used by primary health care workers for early detection of oral cancer in patients with OPMD has not been evaluated fully yet. Mitbander et al [16], in their recent study, have demonstrated the potential use of multimodal imaging in assisting health care workers in the identification of the need for further evaluation in patients with oral lesions. The provision of such diagnostic adjuncts in the hands of frontline health care workers, dentists, and other primary health care providers can significantly improve the early diagnosis of oral cancer at the community level. Furthermore, the feasibility assessment of these diagnostic adjuncts at the community level will help in designing training protocols, monitoring, and mentoring or supportive supervision plans for the large-scale rollout of oral cancer screening. The noninvasive adjuncts are not meant to replace oral biopsy but rather to guide the clinician in selecting the patients with OPMD to undergo biopsy and also to guide the best possible lesional area to biopsy. This intervention can help in making an impact on the oral cancer control efforts in India. An ancillary outcome of this study may be the estimation of the quit rate of tobacco andareca nut habits among patients with OPMD.

We are likely to face challenges in ensuring cessation of the tobacco, areca nut, and alcohol habits by the individuals enrolled in this study due to the deep-rooted social acceptance of these habits. The lack of oral cancer awareness among the general population may also pose a hindrance to the successful conduct of the screening camps in the community. However, the involvement of the community stakeholders in the study might assist in overcoming this barrier.

Recently, the consensus guidelines on the management of noncancerous OPMDs have been released. Some of these patients with OPMD have a high chance of progressing to oral cancer in the future. Hence, risk-based classification of these OPMDs becomes imperative to treat patients with a high risk of developing oral cancer at an appropriate early stage to either halt or slow their progression to malignancy. This study will also evaluate risk-based OPMD management protocols for implementation at various levels of health care system. These protocols will help refine the national-level guidelines for OPMD management in noncancerous patients with OPMD.

### Dissemination Plans

The results of this study shall be disseminated through paper presentations in professional conferences and peer-reviewed publications in high-impact scientific journals. In addition, the results shall be shared with the relevant stakeholders and policy makers for potential implementation in a population-wide manner.

## Appendix

Multimedia Appendix 1 Study proforma.

Multimedia Appendix 2 Peer-review comments from the Indian Council of Medical Research - Intramural Project (New Delhi, India).

## Abbreviations

NFHS-5: National Family Health Survey 5
OL: oral leukoplakia
OLP: oral lichen planus
OPMD: oral potentially malignant disorder
OSMF: oral submucous fibrosis
OVE: oral visual examination
TB: toluidine blue
VELscope: Visually Enhanced Lesion scope
