# Supplementary material for: Evaluation of Noninvasive Adjuncts for Early Detection of Oral Cancer in Oral Potentially Malignant Disorders and Development of Risk-Based Management Strategies: Protocol for a Prospective Longitudinal Study
Source: JMIR Res Protoc. 2025 May 28;14:e66285. doi: 10.2196/66285 (PMC12159550; doi:10.2196/66285)
Supplement: Multimedia Appendix 1 [file resprot_v14i1e66285_app1.pdf]

TENTATIVE STUDY PROFORMA

Study Case No. UHID No. Time started:

Date: Age /Sex:

Address:

Phone Number: Marital Status: Occupation:

Education: Monthly income:

History of Present complaint

Onset (mths)		Duration (mths)	
Progress (↑, ↓, ≈)		Pain (VAS Scores 0-10)	
Burning sensation		Bleeding	
Trismus		Swelling	
Dysphagia		Xerostomia	
Paraesthesia		Sharp teeth	
Restorations		Removable prosthesis	
Fixed prosthesis		Local/ Systemic treatment	
Poor oral hygiene		Sun exposure	
Diet low in fruits and vegetables		Alcohol based mouthwash	

Medical History

Diabetes		Immunosuppressive drugs	
Hypertension		HIV/ AIDS	
Thyroid disorders		Previous cancer in self/ spouse	
Liver disorders		Family history of cancer	
Kidney disorders		Inherited conditions	
Cardiovascular disorders		Other systemic diseases	

Oral Habits

	Quantity/use	Frequency/day	Duration /mths	Current status	Quit period
Tobacco					
Gul manjan					
Gutka					
Supari					
Pan					
Pan masala					
Bidi					
Cigarette					
Hookah					
Alcohol					

Fagerstrom Index

0	1	2	3	4	5	6	7	8	9	10

Simplified Oral Hygiene index

**1**	**2**	**3**	**4**	**5**	**6**

Periodontal Screening and Recording code

1	2	3	4

OPMD- Present / Absent

OSMF		Betel quid chewers mucosa		Leukoplakia	
Erythroplakia		Lichen planus		Lichenoid reaction	
PVL		Tobacco pouch keratosis		Smokers palate	

Site of lesion

Unilateral	Right	Left	Bilateral	
Localized			Diffuse	
Buccal	Anterior			Posterior
Labial	Upper			Lower
Commissure	Right			Left
Palate	Anterior			Posterior
Floor of mouth	Anterior			Posterior
Vestibule	Upper			Lower
Gingiva	Upper			Lower
Tongue	Dorsal			Ventral
	Anterior 2/3rd			Posterior 1/3rd
Palate			Uvula	
Retromolar			Pharynx	

ORAL LEUKOPLAKIA

Preleukoplakia	Homogeneous	Non-Homogeneous			Proliferative verrucous
		Speckled	Nodular	Verrucous	

Size Van der waal OLEP clinical

L1 (<2 cm)	L2 (2-4 cm)	L3 (>4cm)	Lx (size not specified)

Oral Lichen Planus/ Lichenoid reactions

Reticular	Linear	Annular	Papular	Plaque	Pigmented	Bullous	Atrophic	Erosive	DSG

Thongsprasom score for OLP

1	2	3	4	5

OSMF Clinical& Functional staging (More 2011)

CLINICAL		FUNCTIONAL	
Stage 1 (S1)		M1 (>35mm)	
Stage 2 (S2)		M2 (25-35mm)	
Stage 3 (S3)		M3 (15-25mm)	
Stage 4(a) (S4a)		M4 (< 15mm)	
Stage 4(b) (S4b)			

Histological features of OPMD

Epithelial Hyperkeratosis		Epithelial Hyperplasia	
Atypia		Mild dysplasia	
Moderate dysplasia		Severe Dysplasia	
Carcinoma in situ			

Oral Cancer (OSCC)

Type

Plaque type	Ulcerative	Exophytic	Endophytic

Tumour Size

TX	T0	Tis	T1	T2	T3	T4a	T4b

Lymph node

NX	N0	N1	N2a	N2b	N2c	N3

Metastases

MX	M0	M1

Histological **Biopsy No:**

Well differentiated		Moderately differentiated	
Poorly differentiated		Vascular invasion	
Perineural invasion			

Stage

0	I	II	III	IVA	IVB	IVC

Conventional Oral Visual Examination: Positive/ Negative

Autoflurescence:	Positive/ Negative
Toludine Blue staining:	Positive/ Negative
Biopsy:	Required/ Not Required
Tobacco and areca nut counseling:	Yes/ No
Mouth self examination:	Yes/ No
Medical management:	Yes/ No
Surgical management:	Yes / No
Patient acceptabilty questionnaire:	Yes/ No
Time finished:	Total time:
Signature :	Date:

This proforma will be used at each follow up
