# Supplementary material for: Evaluation of Noninvasive Adjuncts for Early Detection of Oral Cancer in Oral Potentially Malignant Disorders and Development of Risk-Based Management Strategies: Protocol for a Prospective Longitudinal Study
Source: JMIR Res Protoc. 2025 May 28;14:e66285. doi: 10.2196/66285 (PMC12159550; doi:10.2196/66285)
Supplement: Multimedia Appendix 2 [file resprot_v14i1e66285_app2.pdf]

intramural project

DR Dr R .S Dhaliwal <dhaliwalrs.hq@icmr.gov.in>
Thu, 02 Nov 2023 7:59:35 PM +0530
To "Ruchika Gupta" <ruchika.gupta79@gov.in>
Cc "jyotisharma.uiet" <jyotisharma.uiet@gmail.com>
Tags Forwarded

Dear Ruchika

This is in response to the Intramural project submitted for ICMR Funding. Your project has been shortlisted for funding consideration. Kindly address the following comments and resubmit for further processing and release of funds. Please complete all these activities within the next 3 weeks

PSC Observations:

The researchers aim to test the effectiveness of non-invasive screening adjuncts (Autofluorescence and Toluidine Blue staining) for early detection of cancer in oral potentially malignant disorders. Basis of sample size is not provided. Longitudinal study is proposed; 10 years follow up is needed for effect; institute to give commitment for continuation after sanctioned duration ends. It is not clear whether study is hospital based or population-based. As the patients are tobacco users, the controls should also be tobacco users. Excluding controls with tobacco habits will produce a bias, as tobacco is known cause of cancer. If the objective is to evaluate useful ness of non-screening methods why the epigenetics is included, this has no relation with it. To test and validate risk-specific management protocols for management of patients with OPMDs at primary health care setting. There is no specific protocol for these, will the PI develop them and how? Epidemiologist and statistician to design the study.
Division's Comments:
Well designed study. Long term follow up is required; institute's commitment is required.

Budget justification is requested

Dr.RS Dhaliwal
Scientist G & Head,
Noncommunicable Diseases(NCD)
ICMR, Ansari Nagar,
New Delhi 110029
TeleFax 26588014

डॉ. आर एस धालीवाल
वैज्ञानिक जी एवम अध्यक्ष
असंक्रामक रोग
आई सी एम आर , अंसारी नगर
नई दिल्ली ११००२९
